# Olfactory identification disorders due to Alzheimer’s disease: A new test from France to Quebec

**DOI:** 10.1371/journal.pone.0265764

**Published:** 2022-04-04

**Authors:** Magali Payne, Valeria Manera, Philippe Robert, Clair Vandersteen, Olivier Beauchet, Kevin Galery, Guillaume Sacco, Roxane Fabre, Auriane Gros

**Affiliations:** 1 CoBteK lab (Cognition Behavior and Technology), Université Cote d’Azur, Nice, France; 2 Centre Hospitalier Universitaire de Nice, Service Clinique Gériatrique du Cerveau et du Mouvement, Centre Mémoire Ressources et Recherche, Université Côte d’Azur, Nice, France; 3 Institut Universitaire de la Face et du Cou, Centre Hospitalier Universitaire de Nice, Université Côte d’Azur, Nice, France; 4 Research Center of the Institut Universitaire en Gériatrie de Montréal, University of Montreal, Montreal, Canada; 5 Faculty of Medicine, Department of Medicine, University of Montreal, Montreal, Canada; 6 Division of Geriatric Medicine, Department of Medicine, Sir Mortimer B. Davis Jewish General Hospital and Lady Davis Institute for Medical Research, McGill University, Montreal, Quebec, Canada; 7 Lee Kong Chian School of Medicine, Nanyang Technological University, Singapore, Singapore; 8 Département de Santé Publique, Centre Hospitalier Universitaire de Nice, Université Côte d’Azur Département de Santé Publique, Nice, France; Niigata University, JAPAN

## Abstract

Olfactory identification disorder is regarded as an early marker of Alzheimer’s disease (AD) and of similar diagnostic significance of biological or cognitive markers. Premature damage of the entorhinal olfactory cortex, the hippocampus and the orbitofrontal cortex characterize AD and suggest a specific impairment of olfactory identification. The use of psychophysical olfactory identification tests in clinical diagnostic practice is therefore strongly recommended, but not required. As these widespread tests are rarely used, an innovative test, adapted to this target group has been developed. It has been used and validated in a routine care protocol at different Memory Centers in France and in Quebec, Canada. A total of 157 participants were recruited: including 63 Alzheimer’s patients and 94 healthy controls. The test was composed of 14 odorants diluted into 4 different concentrations. A computer interface generated randomization of 6 odors per participant and the automatic calculation of identification scores, of perceptual thresholds and of composite scores. All participants underwent a Mini Mental Scale Examination within the previous three months or on the same day of the olfactory test. The Alzheimer’s patients had a score between 20 and 30 and healthy controls participants had a score above 28 without any loss of points on recalled items. The results show that our olfactory identification test is able to significantly differentiate Alzheimer’s patients from healthy controls (p < 0.001), and to distinguish the French population tested from the Quebec population (p < 0.001). This study highlights an olfactory identification disorder as a target for early diagnosis of AD. Its cultural qualities make it a potential candidate for differentiated calibration between France and Quebec.

## Introduction

Alzheimer’s disease (AD) is a neurodegenerative disease where olfactory disorders appear prematurely [1] and act as a precursor that precede the clinical phase of the illness It has been shown that low olfactory scores, associated with a lack of awareness of the disorder, are prognostic factors of mild cognitive impairment [2]. According to the DSM V (Diagnostic and Statistical Manual of Mental Disorders), the cognitive deficit associated with AD can be mild (minor neurocognitive disorder) or patent (major neurocognitive disorder) [3]. The degenerative process underlying Alzheimer disease is characterized by the formation of amyloid plaques (Amyloïdopathy) and by the accumulation of neurofibrillary tangles (Tauopathy) [4]. These anatomical lesions are early observed in the transentorhinal and entorhinal regions of the temporal lobe [5] and progress to the limbic areas [6]. During the asymptomatic phase of AD, a gradual degeneration of the primary cortex occurs and then progresses to the hippocampus, the thalamus, the insula and the orbitofrontal cortex [7]. Olfactory disorders worsen as the disease progresses [2]. Early odour identification deficits have been identified [1] as a strong predictor of entry into AD.

Among olfactory disorders, difficulties in olfactory identification are an early marker of vulnerability [8], while the detection threshold remains relatively preserved in early stages of the disease [9]. This is related to an early damage of the entorhinal cortex, hippocampus and orbitofrontal cortex [10–13], with high-level tasks being affected while olfactory information, and thus olfactory threshold is not damaged in the early phase [14,15]. The olfactory identification task involved presenting a set of odors and offering a choice of four to five names for each odor. Scores on olfactory identification tests significantly distinguish Alzheimer’s patients from healthy control [10,11] and could even have a better predictive value than an episodic memory test among adults at risk of cognitive decline [16]. There is a strong correlation between the results of the olfactory identification tests and the cognitive tests [17–20].

Thus, the use of an olfactory test (OT) is recommended [21,22] in daily clinical practice without an isolated use.

It is, in fact, a very good candidate to be used as a marker of the disease in early detection tests [23]. This was demonstrated by Lafaille-Magnan et al. [24].

However, these scientific findings do not necessarily lead to a regular use of olfactory tests [25,26], which could be justified by their time-consuming nature and the lack of consensus on which tests should be used [27].

Many olfactory tests (OT) are available on the market. Some tests are taking into account the cultural dimensions of odors, which is an important aspect of olfactory identification [28], others are multicultural [27].

The best known are: the University of Pennsylvania Smell Identification Test (UPSIT) developed by Doty [29], including short versions [11] and culturally adapted to North American culture, the "Sniffin’ Sticks" test (Burghart Instruments, Wedel, Germany) [30] applicable to European study groups, and the ETOC (European Test of Olfactory Capabilities) which is cross-cultural [31]. All have been evaluated in populations with mild cognitive impairment [32]. They are very rarely used as an adjunct to the premature diagnosis of AD [33].

Olfactory testing in cognitive impairment is commonly performed by otorhinolaryngologists in daily clinical practice or by neurologists or psychiatrists in memory clinics [14,34].

A simple, accurate and inexpensive OT [35] that would minimize the cognitive load [17] is necessary and recommendations have been made to incorporate clinical, cultural and molecular aspects in a test, stipulating the use of odorants of a simple and reproducible molecular structure [27,36].

By taking into account these imperatives, we have created a new OT, the TODA (Computerized Olfactory Test for the Diagnosis of Alzheimer’s Disease), which offers an optimized test through fully automated and computerized processing. This automatization provides reliable and reproductible evaluation [37], as well as time saving, as recommended [25].The soft-touch feel and lightweight design make the touch tablet ergonomic. 14 high-quality fragrances have been manufactured by perfume chemists from the city of Grasse. They were formulated by the Institute of Chemistry of the University of Nice Cote d’Azur.

We used the TODA during memory consultations in 5 different centers in France, and at the Centre of Excellence in Longevity of at RUISSS McGill in Quebec.

The objective of this study was to validate the effectiveness of TODA in mild AD.

Specifically, we aimed to:

- Evaluate the convergence validity of the TODA by comparing scores obtained from the control population and the AD’s population in France and in Quebec.
- Compare results between the control group and the AD group in France and in Quebec.
- Find correlations between the scores obtained in the Mini Mental State Examination (MMSE)(38) and the scores with the TODA.

## Materials and methods

### Design and participants

The study included a total of 157 participants, 63 with mild stage AD and 94 healthy controls (HC). 76 participants were selected from France and 81 from Quebec, Canada. The study population was composed of 42 male and 115 female, with ages ranging from 62 to 95 years (M  =  74.8; SD  =  7.9). All participants were treated in accordance with the ethical guidelines for research provided by the Declaration of Helsinki. The study was approved by the ethics committee “Ile de France X” n° 2019.A00342-55. The data were collected from 2019 to 2021. After signature of informed consent, each participant completed the olfactory test, which lasted about 15 minutes.

### Measurements

AD diagnosis was based on DSM-5 [3] and ICD-10 criteria [38], and was performed by clinicians of the memory centers based on national guidelines, including scores a MMSE (between 20 and 30, corresponding to minor or slight to moderate major neurocognitive disorder), neuropsychological testing, MRI +/- FDG-PET and lumbar puncture. Biomarker data was not available. HC subjects had no previous history of neurological disorders, and were screened using the MMSE (no point lost on the recall items). For all subjects, the MMSE [39] test was carried out over the three months preceding the olfactory evaluation. Demographic information is reported in Table 1.

**Table 1:** Participants characteristics in France and Quebec, age, gender, diagnosis and mini mental state evaluation.

**Population n = 157**									
** **	** **	** **	** **	**France n = 76**			**Quebec n = 81 **		
	**mean**	**[SD]**	**Median**	**mean**	**[SD]**	**Median**	**mean**	**[SD]**	**Median**
**Age**	74.8	[7.9]	74.0	74.9	[6.6]	75.0	74.7	[9.1]	74.0
**MMSE** ^**a**^	27.2	[3.0]	28.0	27.5	[2.8]	29.0	27.0	[3.2]	28.0
	**n**	**(%)**	** **	**n**	**(%)**	** **	**n**	**(%)**	
**Diagnostic**			** **						
HC^b^	94	(59.9)		45	(59.2)		49	(60.5)	
AD^c^	63	(38.2)		31	(40.8)		32	(39.5)	
** **	** **	** **	** **	**HC** ^**b**^ **n = 94**			**AD n = 63**		
	**n**	**(%)**	** **	**n**	**(%)**	** **	**n**	**(%)**	
**Gender**									
Female	115	(73.2)		79	(84.0)		36	(57.1)	
Male	42	(26.8)		15	(16.0)		27	(42.9)	

Table 1.

^a^ MMSE = Mini Mental Scale Evaluation score.

^b^ HC = Healthy Control subjects.

^c^ AD = subjects with minor or slight to moderate major neurocognitive disorder on the MMSE scale and a diagnosis of Alzheimer’ disease.

### Materials

#### Odors

Odors come in a case, manufactured in accordance with EU regulation 2017–745 as a class 1 medical devices. This odorant case is easily transportable and hermetically sealed.

Fragrant compositions are diluted to any desired concentration in a range from 1 to 40% and are mixed with paraffin wax to be converted in solid form. The odorants are formulated in accordance with the standards of International of Fragrance Association (IFRA) recommendations. A solid form has been favored in order to adapt to an older public, and to avoid the diffusion of micro-droplets in hospital environment. Each scent is presented in a compact jar and is available in four concentrations (which depends on the detection threshold). Concentration 1 varied between 1 and 5% of olfactory raw material, concentration 2 was at 10%, concentration 3 at 20% and concentration 4 at 40%.

The following scents were used: citrus, wood, chocolate, strawberry, grass, mint, coconut, clean, rose, vanilla, almond, jasmine, lavender and pear. These 14 scents were selected according to their perceptual aspects: pleasant, familiar, fruity, floral, food or natural odor, trigeminal or cultural.

#### Computerized olfactory test

This computerized version has been developed as a web application accessible via Internet and available on computer, tablets or mobile phones. During the test, the answers are recorded and the scores are automatically calculated, allowing the clinician to focus on the patient. This application can also be used off-line.

**Fig 1:**
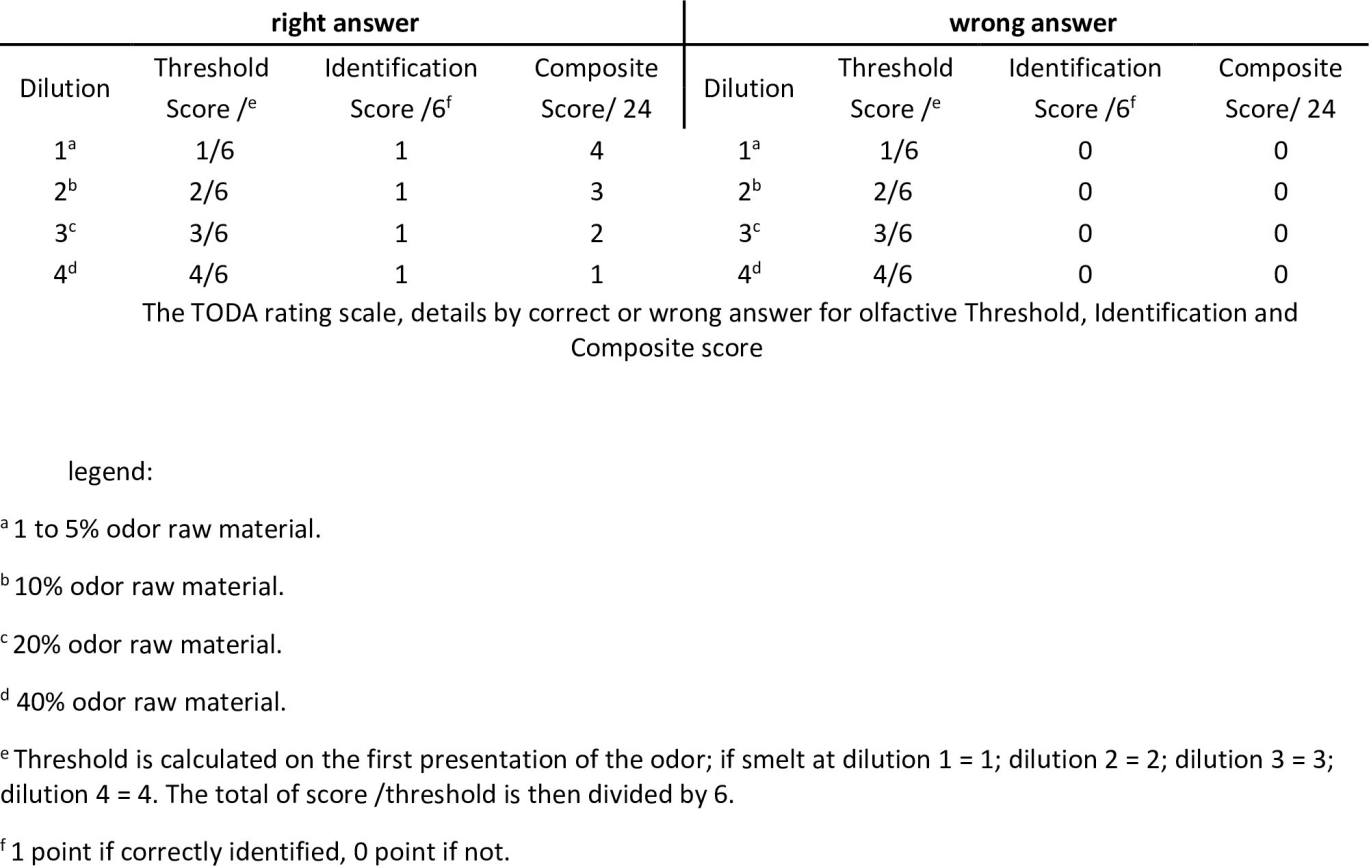
The TODA rating scale.

The computerized olfactory test is able to automatically calculate three scores (Fig 1).

- Identification: the number of correctly identified odors. The total score is based on 6 points.
- Threshold: average intensity at which the subject perceived the odor. The total score is based on 4 levels.
- Composite: combination of olfactive Identification and Threshold results. A point is awarded when an odor is correctly recognized, and more points are attributed for increasingly low concentrations: 4 points at concentration of 1, 3 points at concentration of 2, 2 points at concentration of 3 and 1 point at concentration of 4. No points are awarded for a wrong answer. The total score is based on 24 points.

#### Test procedure

The computerized sequence displays simple instructions to follow: "you will smell an odor and at your signal, you will identify it among 4 images. If you do not smell the odor, a higher concentration will be presented to you”.

Images are provided for identification in order to avoid bias due to a lack of words, that is frequent in early stages of AD.

**Fig 2:**
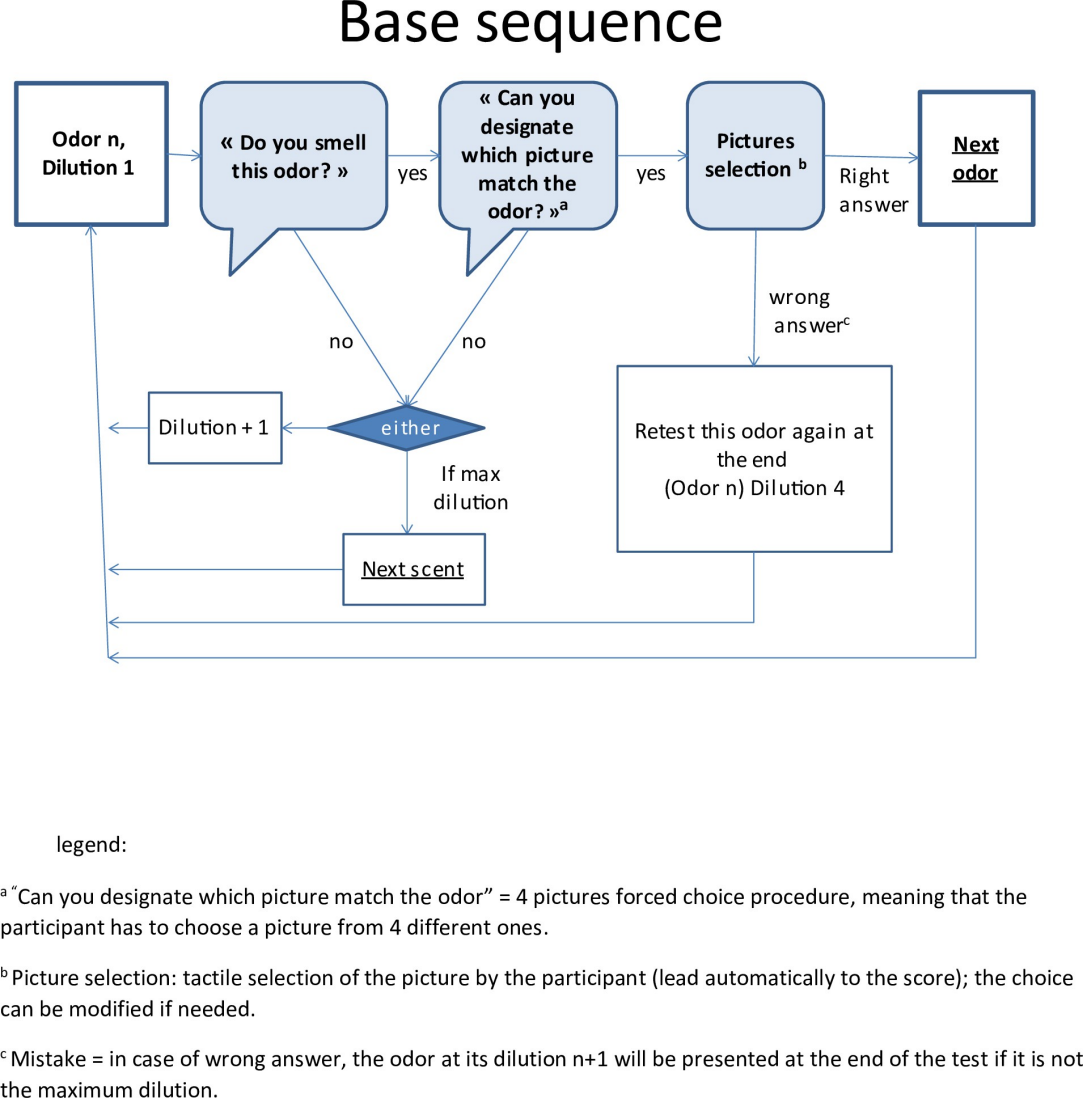
TODA’s computerized base sequence.

Only six odors are randomly selected to respect the format of the test and to avoid the saturation on smell perception. Each scent jar was identified by a single letter code (unrelated to the odorant), to indicate which scent should be tested by the participant. Coding with letters of the alphabet (unlike colors frequently used in other tests) ensures that participants are not influenced. The computerized sequence follows a defined flow (Fig 2).

#### Statistical analysis

Quantitative variables were described using the mean and standard deviation and frequency and percentage for categorical variables. Data were compared between HC and AD groups, and between the France and Quebec groups. The student t-test, or Mann-Whitney test were used for quantitative variables based on the nature of variables. Chi-square test or Fisher’s exact test were used for the categorical variables according to the distribution of the population. Factors associated with the identification score as well as the composite score were assessed. Pearson correlation coefficients and Student’s t-tests were used to assess age, gender, diagnosis and country of origin. Multivariate analysis was performed using linear regression with outcome variables identification and composite scores and age, test center and diagnosis as covariates. P-values <0.05 were considered significant in all the analyses and 95% confidence intervals (95% CI) were reported. All statistical analyses were performed using R 4.0.5 software.

## Results

### Olfactory test validity

In France, HC and AD subjects are significantly differentiate by the olfactive Identification score (p = 0,005), and very significantly by the Composite score (p<,001), with Threshold scores non-significant (p = 0,032). In Quebec, HC and AD subjects are significantly differentiated by the Olfactive Identification score (p < 0.001) and the Composite score (p < 0.001). The detection threshold score is non-significant (p = 0.985).

The identification scores between French and Quebec HC (p = 0,827) and AD subjects (p = 0,111) are non-significant, as well as the Threshold scores (respectively p = 0,062 and p = 0,820).

The composite score is significantly different in healthy controls (HC) subjects between France and Quebec (p<0.001) but non- significantly between in AD subjects between France and Quebec (p = 0,113). The results of olfactory scores comparison between France and Quebec are shown in Table 2.

**Table 2:** Olfactory scores comparison between Quebec and France.

		France—n = 76			Quebec—n = 81			
		mean	SD	p-value*	mean	SD	p-value**	p-value***
AGE	HC	73.9	[5.8]	0.128	71.8	[8.4]	**< .001**	0.295
	AD	76.3	[7.5]		79.1	[8.5]		0.166
MMSE	HC	29.3	[0.7]	**< .001**	29.3	[0.8]	**< .001**	0.966
	AD	24.7	[2.3]		23.4	[2.1]		**0.019**
IDENTIF.^a^	HC	4.5	[1.3]	**0.005**	4.6	[1.2]	**< .001**	0.827
	AD	3.7	[1.3]		3.1	[1.7]		0.111
THRESHOLD^b^	HC	1.0	[0.1]	0.032	1.1	[0.2]	0.465	0.062
	AD	1.2	[0.3]		1.2	[0.4]		0.820
COMPOSITE^c^	HC	17.8	[4.6]	**< .001**	13.9	[4.9]	**< .001**	**< .001**
	AD	10.7	[5.1]		8.7	[4.9]		0.113

*p-value comparisons scores for french AD and HC.

**p-value comparisons scores for Quebec AD and HC.

***p-value comparison for AD and HC for France and Quebec population.

Table 2.

^a^ IDENTIF. = Identification score.

^b^ THRESHOLD = Threshold score.

^c^ COMPOSITE = combination of Identification and Threshold score (Fig 1).

“We calculated multivariate models on the identification score and the composite score to control for effects of age and test center. The results showed that the scores remain significantly lower for AD subjects compared to HC subjects for identification scores (Coeff Adj = -1.21, 95% CI = [-1.67; 0.75], p < .001 and composite scores (Coeff Adj = -6.11, 95% CI = [-7.77; 4.46], p < .001) after controlling for age and test center (see Table 3)”.

**Table 3:** Factors associates to the identification and composite scores.

	Identification			Composite		
	Adj Coeff	[95% CI]	p-value	Adj Coeff	[95% CI]	p-value
Age	0.00	[-0.03; 0.03]	0.924	-0.01	[-0.11; 0.10]	0.888
	Adj Coeff	[95% CI]	p-value	Adj Coeff	[95% CI]	p-value
**Center**						
France	ref			ref		
Quebec	-2.22	[-0.65; 0.22]	0.326	-3.13	[-4.68; -1.58]	< .001
**Diagnosis**						
HC^a^	ref			ref		
AD^b^	-1.21	[-1.67; -0.75]	< .001	-6.11	[-7.77; 4.46]	< .001

^a^ HC = Healthy Control subjects.

^b^ AD = subjects with minor or slight to moderate major neurocognitive disorder on the MMSE scale and a diagnosis of Alzheimer’ disease.

#### Correlation between olfactory score and MMSE score

**Fig 3:**
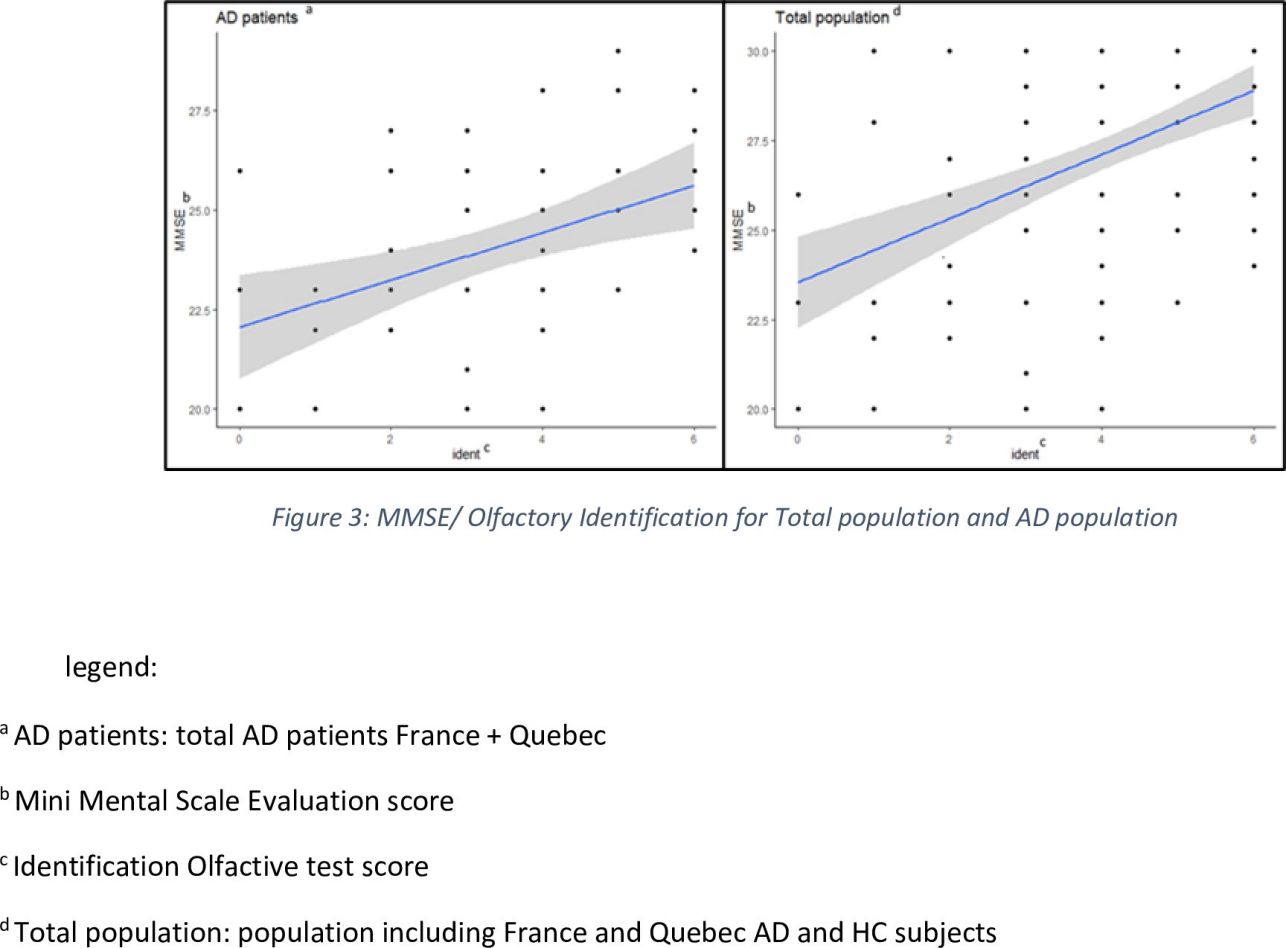
Correlations MMSE/Olfactory identification.

For the total population, our findings show a poor but positive correlation between the MMSE score and the identification score using the Spearman’s rho coefficient (r = 0.33; p < .001), and between the MMSE score and the Composite score (r = 0.42, p < .001), and no correlation at all between MMSE score and the Threshold score (r = -0.06; p = 0.495) (see Fig 3).

When we investigated these correlations by pathology, there was no significance in AD groups in France and Quebec with a non-significant correlation MMSE-odor identification (r = 0.38, p = 0.002) and composite score (r = 0,41, p = 0,001). We observed a negative correlation for the HC group of both populations, with a relation MMSE- odor identification r = -0.28 (p = 0.007) and MMSE- composite scores of r = -0.25 (p = 0.018).

## Discussion

This study highlights the importance of using olfactory tests in clinical practice for early diagnosis of AD. For this reason, the psychophysical olfactory test TODA was developed. Our objectives were to compare the olfactory results obtained from the AD and HC groups in France and Quebec and to investigate correlations between cognitive scores on MMSE and TODA. Olfactory scores on TODA significantly differentiated AD patients from controls within French and Quebec population. This is consistent with previous studies [16,35] and highlights the olfactory identification task as a true biomarker of AD. In accordance with the literature [25,40,41], our results demonstrated that the olfactory test is a valid and reliable tool for the diagnosis of AD. As we know, the accuracy of such tests is comparable to measurements obtained by neuroimaging and cerebrospinal fluid biomarkers, which makes it a reliable biomarker [16]. The olfactory composite results with the TODA also differentiated the AD group from HC within each population, as highlighted for Quebec [20] and France [32].

The absence of correlation between the AD subjects and the HC subjects in France and Quebec can’t be assigned to MMSE or age. It can rather be attributed to the importance of cultural aspects in olfaction. The TODA allows to establish a cultural differentiation because of the choice of the odorants used in the test. Studies demonstrated the effect of culture on olfactory identification, and a variability in olfactory identification secondary to the culture of origin [28,42]. This highlights the need for specific calibration in France and Canada as it has been done for the UPSIT in different countries [40,41,43]. As in the TODA, the choice was to integrate cultural odors and consequently to identify the odors presenting the most significant differences in the two populations

This study showed no connection between olfactory perception threshold and AD, which is expected in the early phase of the disease that is characterized by central damage [2,14]. Regarding the correlation of results between MMSE and olfactory scores, our findings indicated significance in AD patients and HC, which is the result of a strong correlation already established between MMSE scores and olfactory identification scores [9]. Olfactory identification performance diminishes as the MMSE score decreases [19,34,44] given to the association of olfactory identification and cognitive performance in AD. These results highlight also the relationship between olfactory dysfunction and cognitive condition in elderly people and the usefulness of TODA test as an additional marker of age- and pathology-related cognitive decline, or to detect persons at risk of cognitive decline. Indeed, it has been shown that olfactory changes could better predict cognitive decline compared to neuropsychological measures [45–47].

In addition, recent findings showed that, among other sensory markers (vision and audition), olfaction was identified as the most robust predictor of cognitive status and decline and, more specifically, the sensory modality with the greatest predictive power with respect to changes in episodic memory, digit symbol substitution, and vocabulary [48].

The MMSE scores of HC, is between 28 and 30 at the MMSE test and AD subjects is between 20 and 30. They do overlap in accordance with the international classifications, and this could explain the difficulty in distinguishing and finding specific correlations by population.

The TODA test could be used as a follow-up for early AD because it is simple to use and quick to perform [9,43], and odors are randomized. That should minimize the re-test effects.

Finally, the TODA test can also be useful for the early and differential diagnosis of dementia with Lewy bodies (DLB). Indeed, recent findings support the possible usefulness of hyposmia as a prodromal biomarker because it is present in some clinically normal GBA mutation carriers [45], is common in idiopathic REM sleep behavior disorder [46,47] and in older adults carrying the APOE ε4 allele, which is the most robust genetic risk allele for AD [49], DLB [50] and DLB spectrum [51] independently of AD pathology [52]. In addition, APOE-ε4 being linked to hippocampal atrophy and learning/memory phenotypes across the AD/DLB spectrum [53], it highlights once more the relationship between olfactory dysfunction and cognitive condition.

Furthermore, studies suggest that olfactory function tests may be useful in differentiating DLB from AD [47,54–56].

Finally, TODA test in association with other markers (such as motor markers) may be employed to perform early, differential diagnosis and therapeutic strategies in AD and DLB.

There are limitations in this study that should be addressed. First of all, the correlation was only made with a global cognitive measure (MMSE). This is the consequence of a choice based on the total duration of the evaluation that we wished short. Indeed, longer and more specific cognitive assessments will require a long waiting time in clinical practices. Also, it would have been interesting to allow the olfactory testing associated with the MMSE to be used as a quick screening to use as soon as possible while waiting for longer neuropsychological consultations. For the time being, it is necessary to combine this test with an in-depth examination of neuropsychological abilities [17], or with other cognitive variables which also influence olfactory scores [44] such as working memory, verbal fluency and executive functions [34] or naming and semantic memory [21].

The reference population came from hospital memory consultations to investigate mild cognitive impairments and probable AD. They are not exactly representative of the global early AD population and in a way that they had higher cognitive scores, which reduces the possibility of correlation by pathology and by country.

The TODA needs to be calibrated for every country in order to distinguish the cultural specificity of each odor, and the retest effect to be evaluate.

## Conclusion

Our olfactory test has proven its validity to significantly differentiate AD patients from HC and can therefore be useful as a diagnostic aid. The significantly different scores in France and Quebec encourage country-specific calibration. Finally, because of its correlation with the MMSE, this olfactory test could also be useful as a measure for monitoring the evolution of AD at early stage.

## Supporting information

S1 Raw data File containing all data underlying findings for the study. (XLSX)

## References

[1] Devanand DP, Michaels-Marston KS, Liu X, Pelton GH, Padilla M, Marder K, et al. Olfactory Deficits in Patients With Mild Cognitive Impairment Predict Alzheimer’s Disease at Follow-Up. Am J Psychiatry. 2000 Sep;157(9):1399–405. doi: 10.1176/appi.ajp.157.9.1399 10964854

[2] Demarquay G, Ryvlin P, Royet JP. Olfaction et pathologies neurologiques : revue de la littérature. Rev Neurol (Paris). 2007 Feb;163(2):155–67. doi: 10.1016/s0035-3787(07)90387-2 17351535

[3] Association AP. DSM-5 : diagnostic and statistical manual of mental disorders. 5°. American Psychiatric Association

[4] Braak Braak, Jurgen. Staging of Alzheimer-Related cortical destruction. Eur Neurol. 1993;(33):403–8. doi: 10.1159/000116984 8307060

[5] Wang J, Eslinger PJ, Doty RL, Grunfeld R, Sun X, Connor JR, et al. Olfactory Deficit Detected by fMRI in Early Alzheimer’s Disease. 2012;20. 10.1016/j.brainres.2010.08.018 PMC3515873 20709038

[6] Braak H, Tredici KD. Where, when, and in what form does sporadic Alzheimer’s disease begin? 2012;25(6):7–7. 10.1097/WCO.0b013e32835a3432 23160422

[7] Barresi M, Ciurleo R, Giacoppo S, Foti Cuzzola V, Celi D, Bramanti P, Marino S. Evaluation of olfactory dysfunction in neurodegenerative diseases. J Neurol Sci. 2012 Dec;323(1–2):16–24. doi: 10.1016/j.jns.2012.08.028 23010543

[8] Sun GH, Raji CA, MacEachern MP, Burke JF. Olfactory identification testing as a predictor of the development of Alzheimer’s dementia: A systematic review. The Laryngoscope. 2012 Jul;122(7):1455–62. doi: 10.1002/lary.23365 22552846

[9] Serby M, Larson P, Kalskein D. The nature and course of olfactory deficits in Alzheimer’s disease. Am J Psychiatry. 1991;(148):357–60. doi: 10.1176/ajp.148.3.357 1992839

[10] Doty Reyes, Gregor. Presence of Both Odor Identification and Detection Deficits in Alzheimer’ s Disease’. Brain Res Bull. 1987;(18):597–600. 10.1016/0361-9230(87)90129-8 3607528

[11] Tabert MH, Liu X, Doty RL, Serby M, Zamora D, Pelton GH, et al. A 10-item smell identification scale related to risk for Alzheimer’s disease. Ann Neurol. 2005;58(1):155–60. doi: 10.1002/ana.20533 15984022

[12] Wilson RS, Schneider JA, Arnold SE, Tang Y, Boyle PA, Bennett DA. Olfactory Identification and Incidence of Mild Cognitive Impairment in Older Age. Arch Gen Psychiatry. 2007 Jul 1;64(7):802. doi: 10.1001/archpsyc.64.7.802 17606814

[13] Wilson RS, Arnold SE, Schneider JA, Boyle PA, Buchman AS, Bennett DA. Olfactory Impairment in Presymptomatic Alzheimer’s Disease. Ann N Y Acad Sci. 2009 Jul;1170(1):730–5. doi: 10.1111/j.1749-6632.2009.04013.x 19686220 PMC2857767

[14] Marin. Differential diagnosis and classification of Apathy. Am J Psychiatry. 1990;(147). doi: 10.1176/ajp.147.1.22 2403472

[15] Rahayel S, Frasnelli J, Joubert S. The effect of Alzheimer’s disease and Parkinson’s disease on olfaction: A meta-analysis. Behav Brain Res. 2012 May;231(1):60–74. doi: 10.1016/j.bbr.2012.02.047 22414849

[16] Devanand DP. Olfactory Identification Deficits, Cognitive Decline, and Dementia in Older Adults. Am J Geriatr Psychiatry. 2016;24(12):1151–7. doi: 10.1016/j.jagp.2016.08.010 27745824 PMC5136312

[17] Hedner M, Larsson M, Arnold N, Zucco GM, Hummel T. Cognitive factors in odor detection, odor discrimination, and odor identification tasks. J Clin Exp Neuropsychol. 2010 Dec 8;32(10):1062–7. doi: 10.1080/13803391003683070 20437286

[18] Larsson M, Finkel D, Pedersen NL. Odor Identification: Influences of Age, Gender, Cognition, and Personality. J Gerontol B Psychol Sci Soc Sci. 2000 Sep 1;55(5):P304–10. doi: 10.1093/geronb/55.5.p304 10985295

[19] Velayudhan L, Pritchard M, Powell JF, Proitsi P, Lovestone S. Smell identification function as a severity and progression marker in Alzheimer’s disease. Int Psychogeriatr. 2013 Jul;25(7):1157–66. doi: 10.1017/S1041610213000446 23597130

[20] Djordjevic J, Jones-Gotman M, De Sousa K, Chertkow H. Olfaction in patients with mild cognitive impairment and Alzheimer’s disease. Neurobiol Aging. 2008 May;29(5):693–706. doi: 10.1016/j.neurobiolaging.2006.11.014 17207898

[21] Naudin M, Mondon K, Atanasova B. Alzheimer’s disease and olfaction. Gériatrie Psychol Neuropsychiatr Viellissement. 2013;(3):287–93. doi: 10.1684/pnv.2013.0418 24026131

[22] Fusetti M, Fioretti AB, Silvagni F, Simaskou M, Sucapane P, Eibenstein A. Smell and preclinical Alzheimer disease: study of 29 patients with amnesic mild cognitive impairment. J Otolaryngol Head Neck Surg. 2010 Apr;175–81. 20211105

[23] Velayudhan L. Smell identification function and Alzheimer’s disease: A selective review. Curr Opin Psychiatry. 2015 Mar;28(2):173–9. doi: 10.1097/YCO.0000000000000146 25594420

[24] Lafaille-Magnan M-E, Poirier J, Etienne P, Tremblay-Mercier J, Frenette J, Rosa-Neto P, et al. Odor identification as a biomarker of preclinical AD in older adults at risk. Neurology. 2017 Jul 25;89(4):327–35. doi: 10.1212/WNL.0000000000004159 28659431 PMC5574678

[25] Eibenstein A, Fioretti AB, Lena C, Rosati N, Amabile G, Fusetti M. Modern psychophysical tests to assess olfactory function. Neurol Sci. 2005 Jul;26(3):147–55. doi: 10.1007/s10072-005-0452-3 16086127

[26] Lombion-Pouthier S, Vandel P, Nezelof S, Haffen E, Millot J-L. Odor perception in patients with mood disorders. J Affect Disord. 2006 Feb;90(2–3):187–91. doi: 10.1016/j.jad.2005.11.012 16376994

[27] Gros A, Manera V, De March CA, Guevara N, König A, Friedman L, et al. Olfactory disturbances in ageing with and without dementia: towards new diagnostic tools. J Laryngol Otol. 2017 Jul;131(7):572–9. doi: 10.1017/S0022215117000858 28424103

[28] Chrea C, Valentin D, Abdi H. Graded Structure in Odour Categories: A Cross-Cultural Case Study. Perception. 2009 Feb;38(2):292–309. doi: 10.1068/p5687 19400437

[29] Doty Shaman, Dann Doty, Shaman Development. Development of the University of Pennsylvania Smell Identification Test: A Standardized Microencapsulated Test of Olfactory Function. 1984 p. 489–502. (Physiology & Behavior; vol. 32). 6463130 10.1016/0031-9384(84)90269-5

[30] Hummel T, Kobal G, Gudziol H, Mackay-Sim A. Normative data for the “Sniffin’ Sticks” including tests of odor identification, odor discrimination, and olfactory thresholds: an upgrade based on a group of more than 3,000 subjects. Eur Arch Otorhinolaryngol. 2007 Feb 2;264(3):237–43. doi: 10.1007/s00405-006-0173-0 17021776

[31] Thomas-Danguin T, Rouby C, Sicard G, Vigouroux M, Farget V, Johanson A, et al. Development of the ETOC: A European Test of Olfactory Capabilities. Rhinol J. 2003;10142–51. 14579654

[32] Joussain P, Rouby C, Bessy M, Bensafi M. The European Test of Olfactory Capabilities (ETOC): Sensitivity to Pathologies, Age, Culture and Gender. 2014;6.

[33] Rumeau C, Nguyen DT, Jankowski R. Comment tester l’olfaction avec le Sniffin’ Sticks test®. Ann Fr Oto-Rhino-Laryngol Pathol Cervico-Faciale. 2016 Jun;133(3):183–6. 10.1016/j.anorl.2015.08.004 26344139

[34] Reden J, Draf C, Frank RA, Hummel T. Comparison of clinical tests of olfactory function. Eur Arch Otorhinolaryngol. 2016 Apr;273(4):927–31. doi: 10.1007/s00405-015-3682-x 26050222

[35] Velayudhan L. Smell identification function and Alzheimerʼs disease: a selective review. Curr Opin Psychiatry. 2015 Jan;1. doi: 10.1097/YCO.0000000000000146 25594420

[36] Gros A, Payne M. Olfactory Test Contributions in the Diagnosis and Follow-Up of MCI and MA Patients. Biomed J Sci Tech Res [Internet]. 2018;9(1). Available from: https://biomedres.us/fulltexts/BJSTR.MS.ID.001730.php.

[37] Gros Bensamoun, Manera Fabre, Zacconi-Cauvin Thummler, Benoit Robert, David. Recommendations for the Use of ICT in Elderly Populations with Affective Disorders. Front Aging Neurosci [Internet]. 2016 Nov 8 [cited 2020 Feb 19];8. Available from: doi: 10.3389/fnagi.2016.00269 27877126 PMC5099137

[38] Organisation Mondiale de la Santé. CIM-10 Classification Internationale des Maladies et des Troubles Mentaux et troubles du Comportement, Critères Diagnostiques pour la Recherche. Masson; 1994.

[39] Folstein MF, Folstein SE, McHugh PR. “Mini-mental state”. J Psychiatr Res. 1975 Nov;12(3):189–98. doi: 10.1016/0022-3956(75)90026-6 1202204

[40] Mackay-Sim A, Doty RL. THE UNIVERSITY OF PENNSYLVANIA SMELL IDENTIFICATION TEST: NORMATIVE ADJUSTMENT FOR AUSTRALIAN SUBJECTS. AUSTRALIAN JOURNAL OF OTOLARYNGOLOGY. 2001.

[41] Liu H. Performance on a smell screening test (The MODSIT): A study of 510 predominantly illiterate Chinese subjects. Physiol Behav. 1995 Dec;58(6):1251–5. doi: 10.1016/0031-9384(95)02042-x 8623028

[42] Chrea C, Valentin D, Sulmont-Rossé C, Ly Mai H, Hoang Nguyen D, Abdi H. Culture and odor categorization: agreement between cultures depends upon the odors. Food Qual Prefer. 2004 Oct;15(7–8):669–79.

[43] Ogihara H, Kobayashi M, Nishida K, Kitano M, Takeuchi K. Applicability of the Cross-Culturally Modified University of Pennsylvania Smell Identification Test in a Japanese Population. Am J Rhinol Allergy. 2011 Nov;25(6):404–10. doi: 10.2500/ajra.2011.25.3658 22185745

[44] Roberts RO, Christianson TJH, Kremers WK, Mielke MM, Machulda MM, Vassilaki M, et al. Association Between Olfactory Dysfunction and Amnestic Mild Cognitive Impairment and Alzheimer Disease Dementia. JAMA Neurol. 2016 Jan 1;73(1):93. doi: 10.1001/jamaneurol.2015.2952 26569387 PMC4710557

[45] Devanand DP, Tabert MH, Cuasay K, Manly JJ, Schupf N, Brickman AM, et al. Olfactory identification deficits and MCI in a multi-ethnic elderly community sample. Neurobiol Aging. 2010 Sep;31(9):1593–600. doi: 10.1016/j.neurobiolaging.2008.09.008 18963256 PMC2947189

[46] McKee AC, Au R, Cabral HJ, Kowall NW, Seshadri S, Kubilus CA, et al. Visual Association Pathology in Preclinical Alzheimer Disease. J Neuropathol Exp Neurol. 2006 Jun;65(6):621–30. doi: 10.1097/00005072-200606000-00010 16783172

[47] Westervelt HJ, Bruce JM, Faust MA. Distinguishing Alzheimer’s disease and dementia with Lewy bodies using cognitive and olfactory measures. Neuropsychology. 2016;30(3):304–11. doi: 10.1037/neu0000230 26280301

[48] MacDonald SWS, Keller CJC, Brewster PWH, Dixon RA. Contrasting olfaction, vision, and audition as predictors of cognitive change and impairment in non-demented older adults. Neuropsychology. 2018 May;32(4):450–60. doi: 10.1037/neu0000439 29809033 PMC5975972

[49] Taste Schiffman S., smell and neuropsychological performance of individuals at familial risk for Alzheimer’s disease. Neurobiol Aging. 2002 Jun;23(3):397–404. doi: 10.1016/s0197-4580(01)00337-2 11959402

[50] Shiner T, Mirelman A, Rosenblum Y, Kavé G, Weisz MG, Bar-Shira A, et al. The Effect of GBA Mutations and APOE Polymorphisms on Dementia with Lewy Bodies in Ashkenazi Jews. J Alzheimers Dis. 2021 Apr 6;80(3):1221–9. doi: 10.3233/JAD-201295 33646158 PMC8150431

[51] Tsuang D, Leverenz JB, Lopez OL, Hamilton RL, Bennett DA, Schneider JA, et al. APOE ϵ4 Increases Risk for Dementia in Pure Synucleinopathies. JAMA Neurol. 2013 Feb 1;70(2):223. doi: 10.1001/jamaneurol.2013.600 23407718 PMC3580799

[52] Dickson DW, Heckman MG, Murray ME, Soto AI, Walton RL, Diehl NN, et al. APOE ε4 is associated with severity of Lewy body pathology independent of Alzheimer pathology. Neurology. 2018 Sep 18;91(12):e1182–95. doi: 10.1212/WNL.0000000000006212 30143564 PMC6161556

[53] Saeed U, Mirza SS, MacIntosh BJ, Herrmann N, Keith J, Ramirez J, et al. APOE ‐ε4 associates with hippocampal volume, learning, and memory across the spectrum of Alzheimer’s disease and dementia with Lewy bodies. Alzheimers Dement. 2018 Sep;14(9):1137–47. doi: 10.1016/j.jalz.2018.04.005 29782824

[54] McShane RH. Anosmia in dementia is associated with Lewy bodies rather than Alzheimer’s pathology. J Neurol Neurosurg Psychiatry. 2001 Jun 1;70(6):739–43. doi: 10.1136/jnnp.70.6.739 11385006 PMC1737382

[55] Yoo HS, Jeon S, Chung SJ, Yun M, Lee PH, Sohn YH, et al. Olfactory dysfunction in Alzheimer’s disease–and Lewy body–related cognitive impairment. Alzheimers Dement. 2018 Oct;14(10):1243–52. doi: 10.1016/j.jalz.2018.05.010 29936148

[56] Beach Adler, Zhang Serrano, Sue Driver-Dunckley, et al. Severe hyposmia distinguishes neuropathologically confirmed dementia with Lewy bodies from Alzheimer’s disease dementia. PLOS ONE. 2020. doi: 10.1371/journal.pone.0231720 32320406 PMC7176090

